# Time to Alzheimer's disease diagnosis in Japan: a retrospective observational study

**DOI:** 10.1002/alz70860_097026

**Published:** 2025-12-23

**Authors:** Kensaku Kasuga, Soichiro Shimizu, Noriyuki Kimura, Koji Kasanuki, Shu Sakurai, Shuntaro Serisawa, Teruaki Masuda, Takuya Ataka, Kenji Fuseya, Hiroko Shiina, Yosuke Nakamura

**Affiliations:** ^1^ Niigata University, Niigata, Niigata, Japan; ^2^ Tokyo Medical University, Shinjuku‐ku, Tokyo, Japan; ^3^ Oita University, Yufu, Oita, Japan; ^4^ St. Marianna University School of Medicine, Kawasaki, Kanagawa, Japan; ^5^ Eisai Co., Ltd., Bunkyo‐ku, Tokyo, Japan; ^6^ Eisai Inc., Nutley, NJ, USA

## Abstract

**Background:**

Early diagnosis of Alzheimer's disease (AD) plays an important role in the treatment of the disease; however, little is known about the diagnostic journey of AD patients in Japan. Thus, we conducted a retrospective observational study of patients with AD including those with early‐onset AD (EOAD) to investigate the time from symptom onset to diagnosis, and identify the limiting step within the diagnostic journey in Japan.

**Method:**

Subjects between the ages of 18 and 79 years receiving a diagnosis of “Probable AD dementia”, “MCI due to AD”, or an equivalent diagnosis, and who were diagnosed between April 2011 and March 2023 at participating hospitals across Japan were included in the study. Subjects were categorized by age of onset as EOAD (below 65 years) or late‐onset AD (LOAD, above 65 years). EOAD and LOAD subjects were matched 1:1 by the date of first visit to participating hospitals. EOAD and LOAD subjects were pooled together and used for analysis. Medical information related to diagnoses was extracted from electronic medical records.

**Result:**

A total of 138 subjects with an average ± standard deviation age of onset at 62.8 ± 9.3 years were included in the study. Of these, 85 (61.6%) were female, 95 (68.8%) had an established medical care provider, and 86 (62.3%) had comorbidities under treatment at symptom onset. The time from symptom onset to AD diagnosis was 121.8 ± 88.9 weeks. Especially, the time from symptom onset to the first medical visit was the longest (77.0 ± 74.4 weeks). The time from the first medical visit to the referral visit to a dementia specialist was 34.9 ± 49.2 weeks, and the time from the referral visit to a dementia specialist to diagnosis was 10.5 ± 18.8 weeks.

**Conclusion:**

In Japan, the time from symptom onset to diagnosis takes approximately 2.2 years. The time after symptom onset to reach the initial medical contact consumes the longest time; hence, minimizing this time could shorten the overall diagnostic duration. Since early diagnosis facilitates timely treatment of AD, it is important to raise awareness of the disease and the need for early consultation.

